# Porcine RING Finger Protein 114 Inhibits Classical Swine Fever Virus Replication via K27-Linked Polyubiquitination of Viral NS4B

**DOI:** 10.1128/JVI.01248-19

**Published:** 2019-10-15

**Authors:** Yuexiu Zhang, Huawei Zhang, Guang-Lai Zheng, Qian Yang, Shaoxiong Yu, Jinghan Wang, Su Li, Lian-Feng Li, Hua-Ji Qiu

**Affiliations:** a State Key Laboratory of Veterinary Biotechnology, Harbin Veterinary Research Institute, Chinese Academy of Agricultural Sciences, Harbin, China; Instituto de Biotecnologia/UNAM

**Keywords:** antiviral activity, classical swine fever virus, NS4B protein, RING finger protein 114, ubiquitination

## Abstract

In the host, many RING domain E3 ligases have been reported to inhibit viral replication through various mechanisms. In a previous screen, we found that porcine RING finger protein 114 (pRNF114), a RING domain E3 ubiquitin ligase, inhibits classical swine fever virus (CSFV) replication. This study aimed to clarify the underlying antiviral mechanism of pRNF114 against CSFV. Upon CSFV infection, pRNF114 mRNA was upregulated both *in vitro* and *in vivo*. CSFV replication was significantly suppressed in PK-pRNF114 cells stably expressing pRNF114 by the lentivirus-delivered system, whereas CSFV growth was enhanced in PK-15 cells with RNF114 knockout by the CRISPR/Cas9 system. The RING domain of pRNF114, which has E3 ubiquitin ligase activity, is crucial for its antiviral activity. Mechanistically, pRNF114 interacted with the CSFV NS4B protein through their C-terminal domains, which led to the K27-linked polyubiquitination and degradation of NS4B through a proteasome-dependent pathway. Collectively, these findings indicate that pRNF114 as a critical regulator of CSFV replication and uncover a mechanism by which pRNF114 employs its E3 ubiquitin ligase activity to inhibit CSFV replication.

**IMPORTANCE** Porcine RING finger protein 114 (pRNF114) is a member of the RING domain E3 ligases. In this study, it was shown that pRNF114 is a potential anti-CSFV factor and the anti-CSFV effect of pRNF114 depends on its E3 ligase activity. Notably, pRNF114 targets and catalyzes the K27-linked polyubiquitination of the NS4B protein and then promotes proteasome-dependent degradation of NS4B, inhibiting the replication of CSFV. To our knowledge, pRNF114 is the first E3 ligase to be identified as being involved in anti-CSFV activity, and targeting NS4B could be a crucial route for antiviral development.

## Abstract (precis)

Porcine RING finger protein 114 (pRNF114) is a member of the RING domain E3 ligases. In this study, it was shown that pRNF114 is a potential anti-CSFV factor and the anti-CSFV effect of pRNF114 depends on its E3 ligase activity. Notably, pRNF114 targets and catalyzes the K27-linked polyubiquitination of the NS4B protein and then promotes proteasome-dependent degradation of NS4B, inhibiting the replication of CSFV. To our knowledge, pRNF114 is the first E3 ligase to be identified as being involved in anti-CSFV activity, and targeting NS4B could be a crucial route for antiviral development.

## INTRODUCTION

Classical swine fever, an economically important viral disease of swine, is caused by classical swine fever virus (CSFV), which belongs to the *Pestivirus* genus within the *Flaviviridae* family (1, 2). The viral genome encodes a polyprotein precursor that is further proteolytically cleaved into four structural (C, E^rns^, E1, and E2) and eight nonstructural (N^pro^, p7, NS2, NS3, NS4A, NS4B, NS5A, and NS5B) proteins (3, 4).

CSFV NS4B is a 38-kDa transmembrane protein that consists of 347 amino acids (5). Similar to the case with bovine viral diarrhea virus, another member of the *Pestivirus* genus, the CSFV NS4B protein contains three transmembrane regions (6). NS4B together with other nonstructural proteins (i.e., NS3, NS4A, NS5A, and NS5B) of CSFV forms an RNA replicase complex, which is essential for RNA replication (7–9). The CSFV NS4B protein also possesses nucleoside triphosphatase (NTPase) activity, which is required for CSFV replication. It contains two conserved domains: Walker A (amino acids 209 to 216) and Walker B (amino acids 335 to 342). Walker A is the crucial domain for NTPase activity and RNA replication. Additionally, NS4B is involved in the virulence of CSFV. A study identified a putative Toll/interleukin-1 receptor-like domain on the C-terminal region of NS4B. However, mutation in this domain of NS4B resulted in an attenuated phenotype of a highly virulent Brescia strain of CSFV (10). The exact mechanism of the NS4B-mediated CSFV life cycle and pathogenesis remains elusive. However, it is known that the NS4B protein of flaviviruses modulates the host cell environment to evade host immune responses. The NS4B protein of hepatitis C virus (HCV) can block RIG-I-like receptor (RLR)-mediated interferon signaling by targeting stimulator of interferon genes (STING) and inhibit Toll-like receptor 3 (TLR3)-mediated interferon signaling via inducing TIR domain-containing adaptor inducing IFN-β (TRIF) degradation (11, 12). Recently, CSFV NS4B has been shown to bind with TANK-binding kinase 1 (TBK1) and other 13 host proteins, revealing the functional plasticity of NS4B in virus replication (13).

The RING domain E3 ligases (RING E3s), a group of E3 ligases containing a RING finger domain, are involved in various cellular processes (14–16). In the host, virus replication is extremely regulated by the immune system (17, 18), in which various RING E3s have been implicated (19); hence, the RING E3s may play a pivotal role in regulating virus replication. Accumulating studies have evidenced the important roles of the RING E3s in host responses to viral infections, including directly inhibiting viral replication through interfering with crucial steps of the virus life cycle. MARCH-8 inhibits human immunodeficiency virus type 1 (HIV-1) infection via targeting HIV-1 envelope glycoproteins and reducing their incorporation into the virions (20). TRIM22 and TRIM41 inhibit influenza A virus replication by degrading nucleoprotein in a proteasome-dependent manner (21, 22). TRIM52 targets and degrades the viral NS2A protein to antagonize Japanese encephalitis virus replication (23), and TRIM69 restricts dengue virus (DENV) replication through ubiquitinating the viral NS3 protein (24). The E3 ubiquitin (Ub) ligase Siah-1 ubiquitinates the avian reovirus p10 protein and facilitates proteasomal degradation (25).

Members of the RING ubiquitin-interacting motif (UIM) E3 ligase family, a subfamily of RING E3s, share five highly conserved domains, including a RING domain, a C_2_HC domain, two C_2_H_2_-type zinc fingers, and a UIM-type domain (26). This family contains four members, named RNF114 (also known as ZNF313), RNF125, RNF138, and RNF166. At present, human RNF114 (hRNF114) has been reported to play important roles in the regulation of cell cycle progression, differentiation, and senescence (27, 28). In addition, it also regulates NF-κB activity and T-cell activation (29, 30). However, the antiviral potential of RNF114 has not yet been explored.

Porcine RNF114 (pRNF114) has been screened as a candidate anti-CSFV factor since overexpression of pRNF114 inhibits rCSFV-Fluc (firefly luciferase) replication (31). Moreover, several previous studies have demonstrated that hRNF114 acts as a RING UIM E3 ligase (27, 28, 32). Driven by these facts, we explored the role of pRNF114 in the CSFV replication cycle. In this study, we showed that anti-CSFV function of pRNF114 is determined by E3 ligase activity. Intriguingly, we revealed that pRNF114 directly interacts with viral NS4B protein and results in NS4B protein degradation via a proteasome-dependent pathway. These findings provide new mechanistic insights into the functional annotation of pRNF114 and warrant further studies to exploit these targets as an attractive antiviral.

## RESULTS

### The mRNA transcription level of pRNF114 is upregulated upon CSFV infection.

**FIG 1:**
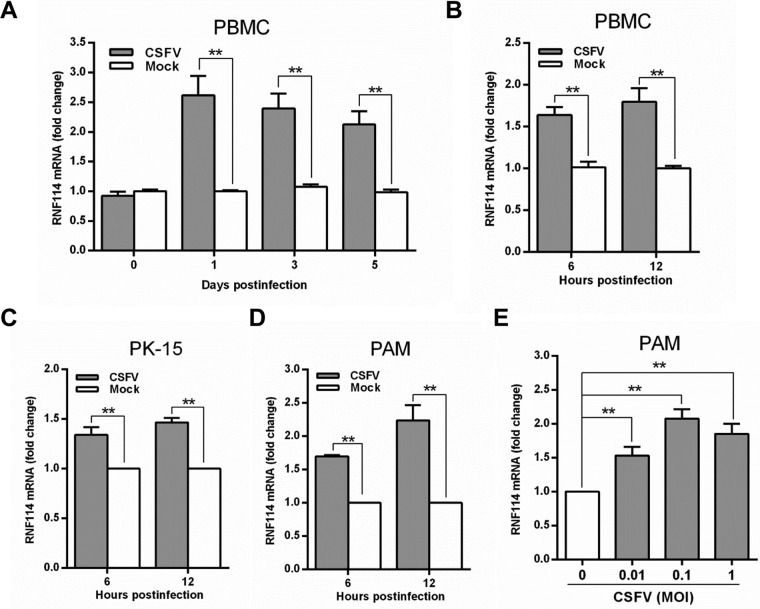
pRNF114 is upregulated upon CSFV infection. (A) The expression of pRNF114 was examined in peripheral blood mononuclear cells (PBMCs) isolated from a CSFV-infected pig at 1, 3, or 5 days postinfection by real-time reverse transcription-quantitative PCR (RT-qPCR). (B to E) Expression of pRNF114 in CSFV-infected PBMCs, PK-15 cells, and porcine alveolar macrophages (PAMs). PBMCs (B), PK-15 cells (C), and PAMs (D) were infected with Shimen (MOI = 0.1), and the expression of pRNF114 was tested at 6 and 12 h postinfection (hpi). (E) The PAMs were infected with Shimen at increasing MOIs and tested at 6 hpi by RT-qPCR. Bars represent SDs. *, *P* < 0.05; **, *P* < 0.01.

To evaluate the effects of pRNF114 during CSFV infection *in vivo* and *in vitro*, we detected the mRNA transcription level of pRNF114 upon CSFV (strain Shimen) infection by a real-time reverse transcription-quantitative PCR (RT-qPCR) assay. The results showed that the pRNF114 mRNA transcription level was increased significantly in peripheral blood mononuclear cells (PBMCs) of CSFV-infected pigs at 1, 3, or 5 days postinfection (Fig. 1A). Consistently, the mRNA transcription level of pRNF114 was increased in the isolated PBMCs (Fig. 1B), PK-15 cells (Fig. 1C), and porcine alveolar macrophages (PAMs) (Fig. 1D) infected with Shimen (multiplicity of infection [MOI] = 0.1) at 6 or 12 h postinfection (hpi), as well as in PAMs at an MOI of 0.01, 0.1, or 1 at 6 hpi (Fig. 1E). These data suggest that CSFV infection can induce the expression of pRNF114, indicating a potential role for pRNF114 in CSFV infection.

### pRNF114 inhibits CSFV replication.

**FIG 2:**
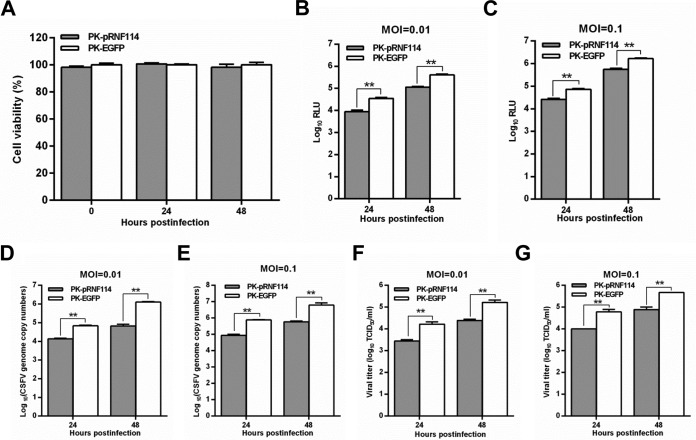
Overexpression of pRNF114 inhibits CSFV replication. (A) Viability of cell lines stably overexpressing pRNF114. (B to G) Influence of overexpression of pRNF114 on CSFV growth. PK-pRNF114 and PK-EGFP cells were infected with rCSFV-Fluc or Shimen (MOI = 0.01 or 0.1) for 24 or 48 h. The luciferase activities were assayed by a luciferase reporter assay system (Promega) and expressed as relative light units (RLU) (B and C). The CSFV genomic RNA copy numbers were quantified by real-time reverse transcription-quantitative PCR (D and E). Viral titers were determined as median tissue culture infective doses (TCID_50_) per milliliter (F and G). Bars represent SDs. *, *P* < 0.05; **, *P* < 0.01.

Considering that pRNF114 potentially inhibits CSFV replication (31), we established PK-pRNF114 or PK-EGFP cells stably expressing pRNF114 or enhanced green fluorescent protein (EGFP) to confirm the antiviral activity of pRNF114 against CSFV. The cell growth and viability of PK-pRNF114 were similar to those of PK-EGFP cells (Fig. 2A). We demonstrated that overexpression of pRNF114 inhibits rCSFV-Fluc replication at an MOI of 0.01 or 0.1 at 24 or 48 hpi (Fig. 2B and C). The anti-CSFV activity of pRNF114 was examined in PK-pRNF114 and PK-EGFP cells upon infection with Shimen (MOI = 0.1 or 0.01). The CSFV RNA (Fig. 2D and E) and the extracellular titers of the progeny virus (Fig. 2F and G) were significantly reduced in PK-pRNF114 cells compared with those in PK-EGFP cells at 24 or 48 hpi.

**FIG 3:**
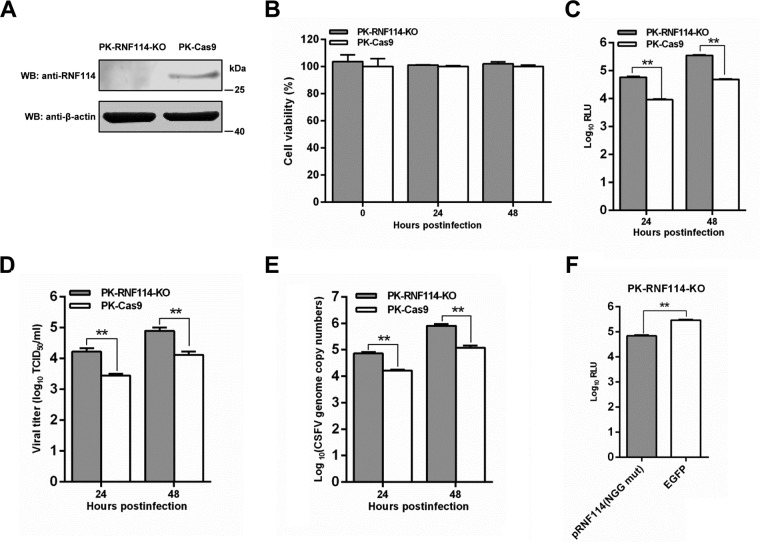
Knockout of RNF114 enhances CSFV replication. (A) Knockout efficiency of RNF114 by the CRISPR/Cas9 system. The protein expression level of RNF114 was examined by Western blotting (WB) in PK-RNF114-KO or PK-Cas9 cells. (B) Viability assay of PK-RNF114-KO and PK-Cas9 cells. (C to E) Influence of knockout of RNF114 on CSFV replication. PK-RNF114-KO and PK-Cas9 cells were infected with the rCSFV-Fluc or Shimen (MOI = 0.01) for 24 or 48 h. The luciferase activities, virus titers, and viral RNA levels were determined by the luciferase reporter assay (C), expressed as median tissue culture infective doses per milliliter (D), and determined by real-time reverse transcription-quantitative PCR (E), respectively. (F) Reconstitution of pRNF114 inhibited CSFV infection. Lenti-EGFP or Lenti-pRNF114(NGG Mut) expressing pRNF114 with the NGG consensus mutation was transduced into PK-RNF114-KO cells, and the cells were then infected with rCSFV-Fluc at an MOI of 0.01 for 48 h. The luciferase activities were measured by luciferase reporter assay. RLU, relative light units. Bars represent SDs. *, *P* < 0.05; **, *P* < 0.01.

To further examine the anti-CSFV effects of pRNF114, we established a PK-RNF114-KO cell line by CRISPR/Cas9 system and used PK-Cas9 as a control. We examined the protein expression level of RNF114 in PK-RNF114-KO and PK-Cas9 cells. The results indicated that RNF114 protein expression was completely abrogated in PK-RNF114-KO cells (Fig. 3A). Subsequently, the replication efficiency of rCSFV-Fluc or Shimen was assessed in PK-RNF114-KO and PK-Cas9 cells. The viability of PK-RNF114-KO cells was indistinguishable from that of PK-Cas9 cells (Fig. 3B). Compared with those in PK-Cas9 cells, the intracellular Fluc activities (Fig. 3C), viral titers (Fig. 3D), and viral RNA copies (Fig. 3E) were increased in PK-RNF114-KO cells. To further demonstrate that the observed phenomenon was not due to off-target effects, Lenti-pRNF114(NGG mut), expressing pRNF114 with the NGG consensus mutation, was transduced into PK-RNF114-KO cells. We found that pRNF114 restoration inhibited CSFV infection compared with that in PK-RNF114-KO cells transduced with Lenti-EGFP (Fig. 3F). Together, these results demonstrate that knockout of RNF114 promotes CSFV replication, suggesting that pRNF114 inhibits CSFV replication.

### E3 ligase activity of pRNF114 is essential for its inhibition of CSFV replication.

**FIG 4:**
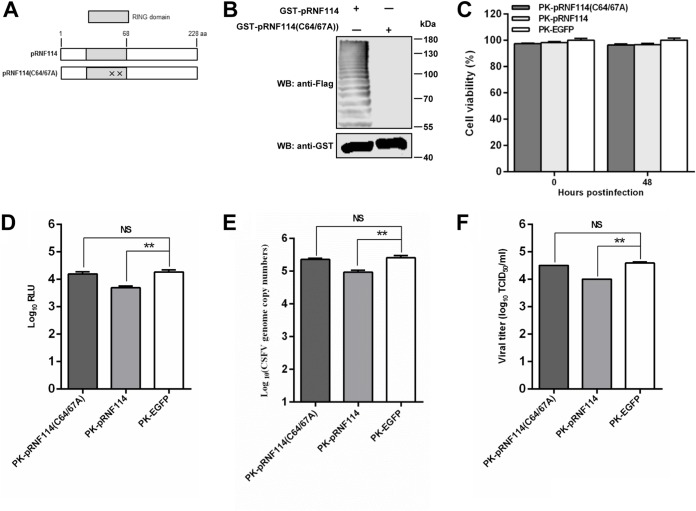
The E3 ligase activity of pRNF114 is essential for its anti-CSFV activity. (A) Schematic structure of pRNF114 and pRNF114(C64/67A). ‘‘X’’ indicates that the cysteine residues at positions 64 and 67 on pRNF114 were replaced with alanine. aa, amino acids. (B) The ubiquitin ligase activity of pRNF114 and pRNF114(C64/67A) was verified by an *in vitro* ubiquitination assay. (C) Viability assay of cell lines stably overexpressing pRNF114, pRNF114(C64/67A), or EGFP. (D to F) Influence of pRNF114(C64/67A) on CSFV replication. The cell lines were infected with rCSFV-Fluc or Shimen (MOI = 0.01) for 48 h. The luciferase activities and genomic copies of CSFV were examined by luciferase reporter assay (D) and real-time reverse transcription-quantitative PCR (E), and titers of CSFV are expressed as median tissue culture infective doses per milliliter (F). Bars represent SDs. NS, not significant. *, *P* < 0.05; **, *P* < 0.01.

The pRNF114 protein belongs to the RING-UIM family, containing a RING domain (19). To investigate whether the E3 ligase activity of pRNF114 is involved in the inhibition of CSFV, we generated a pRNF114 RING domain mutant plasmid, pGST-pRNF114(C64/67A), in which the cysteines (C) at positions 64 and 67 on the pRNF114 RING domain were mutated to alanine (A) (Fig. 4A), and the lack of ubiquitin ligase activity for pRNF114(C64/67A) was verified by an *in vitro* ubiquitination experiment (Fig. 4B). We established PK-pRNF114(C64/67A) cells stably expressing pRNF114(C64/67A), with no difference in cell growth and viability from PK-pRNF114 and PK-EGFP cells (Fig. 4C). The anti-CSFV effects of pRNF114 were detected in those cells. Compared with those in PK-EGFP cells, the intracellular Fluc activities (Fig. 4D), CSFV RNA (Fig. 4E), and viral titers (Fig. 4F) were reduced in PK-pRNF114 cells. In PK-pRNF114(C64/67A) cells, however, pRNF114(C64/67A) failed to display an anti-CSFV action, as demonstrated by higher Fluc activities, viral genome RNA, and virus yields. These data suggest that the anti-CSFV activity of pRNF114 is dependent on the E3 ligase activity.

### pRNF114 interacts with the CSFV NS4B protein.

**FIG 5:**
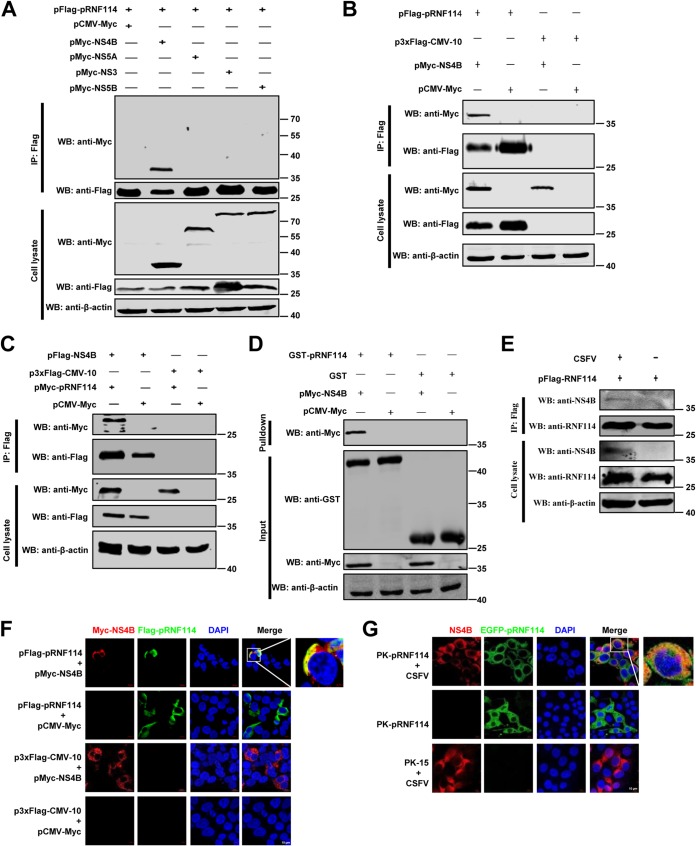
pRNF114 interacts with the CSFV NS4B. (A to C) Exogenous coimmunoprecipitation (co-IP) assay in HEK293T cells. The cells were cotransfected with pFlag-pRNF114 and either pCMV-Myc, pMyc-NS4B, pMyc-NS5A, pMyc-NS3, or pMyc-NS5B for 48 h (A), with pFlag-pRNF114 and either pCMV-Myc or pMyc-NS4B for 48 h (B), or with pFlag-NS4B and either pCMV-Myc or pMyc-pRNF114 for 48 h (C). The cell lysates were subjected to a co-IP assay using anti-Flag MAb and tested using the indicated antibodies by Western blotting. (D) GST pulldown assay. Purified proteins of GST-pRNF114 and GST were incubated with the Myc-NS4B protein. The proteins bound to beads were analyzed by immunoblotting using the indicated antibodies. (E) Endogenous co-IP assay. PK-15 cells transfected with pFlag-pRNF114 were infected with Shimen (MOI = 1) for 48 h and subjected to a co-IP assay using anti-Flag MAb. (F and G) Confocal assay. HEK293T cells were cotransfected with pFlag-pRNF114 and pMyc-NS4B (F), and PK-pRNF114 cells were infected with Shimen (MOI = 1) (G). The cells were subjected to a confocal assay. DAPI, 4′,6-diamidino-2-phenylindole.

Many RING E3s have been reported to restrict the replication of various viruses through interaction with viral nonstructural proteins (21, 23, 24). To clarify the specific targets of pRNF114, we examined the association between pRNF114 and CSFV nonstructural proteins by coimmunoprecipitation (co-IP) and identified NS4B as a pRNF114-interacting partner (Fig. 5A). Furthermore, the interaction between pRNF114 and NS4B was demonstrated by co-IP (Fig. 5B), reciprocal co-IP (Fig. 5C), and glutathione *S*-transferase (GST) pulldown assays (Fig. 5D). Next, we verified the interaction of pRNF114 with NS4B in the context of CSFV infection (Fig. 5E).

To further prove the association pRNF114 and NS4B, we also evaluated whether the pRNF114 protein colocalizes with NS4B in HEK293T cells cotransfected with pFlag-pRNF114 and pMyc-NS4B. The results indicated the colocalization of pRNF114 and NS4B in the cytoplasm (Fig. 5F). We further examined the colocalization of the pRNF114 protein and NS4B in CSFV-infected PK-pRNF114 cells. Confocal images revealed that pRNF114 colocalizes with NS4B in the cytoplasm of the CSFV-infected cells (Fig. 5G). These results together suggest that pRNF114 interacts with the CSFV NS4B protein.

### The pRNF114 C-terminal region interacts with the NS4B C-terminal region.

**FIG 6:**
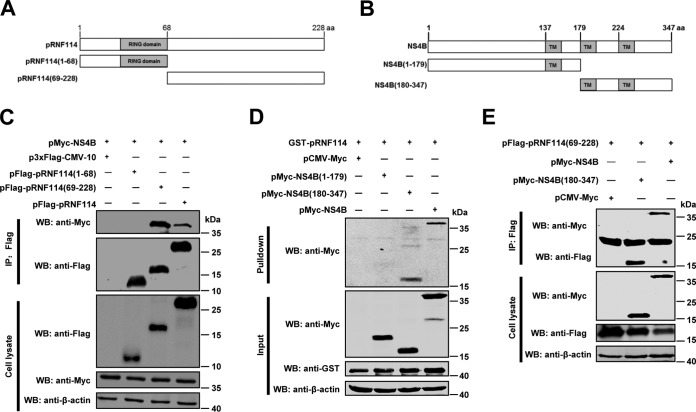
The C-terminal region of pRNF114 interacts with the C-terminal region of NS4B. (A and B) Schematic representations of the domains of pRNF114 (A) and NS4B (B). TM, transmembrane domain. (C) The C-terminal region (aa 69 to 228) of pRNF114 binds to NS4B. HEK293T cell lysates with indicated plasmids were immunoprecipitated with a mouse anti-Flag monoclonal antibody and immunoblotted with the indicated antibodies. (D) The C terminus (aa 180 to 347) of NS4B strongly binds to pRNF114. Lysates of HEK293T cells transfected with the indicated plasmids were pulled down with purified GST-pRNF114 protein. The proteins bound to the beads and the original cell lysates were examined by Western blotting. (E) pRNF114(69-228) is responsible for interacting with NS4B(180-347). Lysates prepared from HEK293T cells cotransfected with the indicated plasmids were immunoprecipitated and immunoblotted with the indicated antibodies.

To further investigate the key regions responsible for the NS4B-pRNF114 interaction, we constructed truncated mutants of pRNF114 and NS4B (Fig. 6A and B). HEK293T cells were transfected with the desired plasmids, and the domain responsible for interaction of pRNF114 with NS4B was determined using co-IP and GST pulldown assays. The results showed that pRNF114(69-228) but not pRNF114(1-68) was involved in the interaction (Fig. 6C) and NS4B(180-347) exhibited a strong interaction with pRNF114 (Fig. 6D). Further results confirmed that NS4B(180-347) interacted with pRNF114(69-228) (Fig. 6E). These findings suggest that the C-terminal region of pRNF114 is necessary for interaction with the C-terminal region of NS4B.

### pRNF114 promotes proteasomal degradation of NS4B.

**FIG 7:**
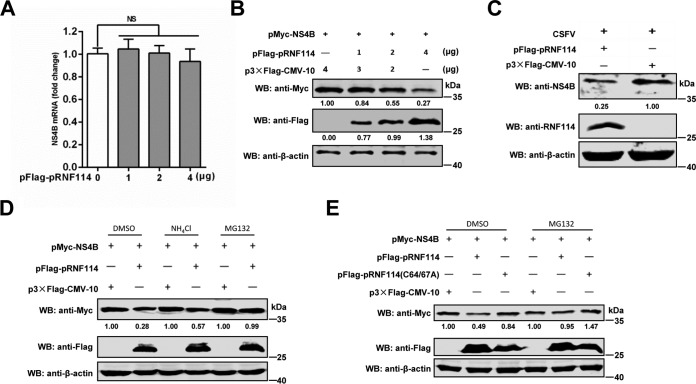
pRNF114 targets NS4B for proteasome degradation. (A to C) NS4B was degraded by pRNF114. HEK293T cells were cotransfected with pMyc-NS4B (1 μg) and increasing doses of pFlag-pRNF114 (0, 1, 2, and 4 μg) into 24-well plates. The expression level of NS4B was detected by real-time reverse transcription-quantitative PCR (A) or Western blotting (B). The PK-15 cells transfected with pFlag-pRNF114 were infected with Shimen (MOI = 1) for 48 h (C). (D) pRNF114 promotes proteasomal degradation of NS4B. pMyc-NS4B was transfected with or without pFlag-pRNF114 into HEK293T cells and then treated for 24 h with dimethyl sulfoxide (DMSO; negative control), MG132 (10 μM), or NH_4_Cl (20 mM). (E) The degradation of NS4B by pRNF114 depends on its E3 ubiquitin ligase. HEK293T cells were cotransfected with pMyc-NS4B and p3×Flag-CMV-10, pFlag-RNF114, or pRNF114(C64/67A) and then were treated for 24 h with DMSO or 10 μM MG132. Cell lysates were then blotted with the indicated antibodies. Results of densitometry analysis to quantify the ratio of NS4B to β-actin are shown at the bottom. Bars represent SDs.

Considering that pRNF114 is an E3 ubiquitin ligase and its enzymatic activity is critical for inhibition of the CSFV replication, we hypothesized that pRNF114 could promote NS4B degradation. To this end, pMyc-NS4B and increasing amounts of pFlag-pRNF114 were cotransfected into HEK293T cells. The results showed that pRNF114 did not influence mRNA transcription (Fig. 7A) but reduced protein expression of NS4B in a dose-dependent manner (Fig. 7B). Upon CSFV infection, the protein expression level of NS4B was decreased in PK-15 cells transfected with pFlag-pRNF114 (Fig. 7C).

There are two major pathways responsible for protein degradation, proteasome pathway and lysosomal proteolysis. To test whether NS4B degradation results from these two pathways, we treated cells with the proteasome inhibitor MG132 or lysosome inhibitor NH_4_Cl. The results showed that pRNF114-induced NS4B degradation was rescued by MG132 but not by NH_4_Cl (Fig. 7D). Next, we determined whether NS4B degradation is dependent on the E3 ubiquitin ligase activity of pRNF114. The results showed that pRNF114(C64/67A) attenuated NS4B degradation when expressed in HEK293T cells compared with the wild-type pRNF114 (Fig. 7E). Collectively, these results indicate that the interaction of pRNF114 with NS4B enhances NS4B degradation in a proteasome pathway, which is dependent on its E3 ubiquitin ligase activity.

### pRNF114 mediates ubiquitination of NS4B.

**FIG 8:**
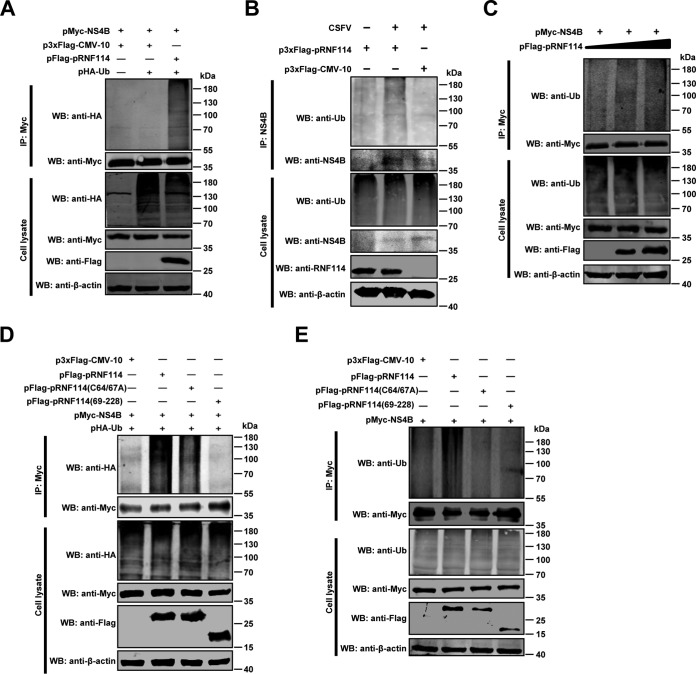
pRNF114 mediates ubiquitination of NS4B. (A and B) The ubiquitination level of NS4B is potentiated by expression of pRNF114. HEK293T cells were cotransfected with the indicated plasmids for 24 h and treated with 10 μM MG132 (Sigma-Aldrich) for an additional 24 h. Cell extracts were prepared and analyzed by co-IP assay using an anti-Myc monoclonal antibody (MAb) followed by Western blotting (WB) with the indicated antibodies (A). PK-15 cells transfected with pFlag-pRNF114 were infected with Shimen (MOI = 1) for 48 h. Lysates derived from these cells were subjected to IP using anti-NS4B polyclonal antibody and WB with the indicated antibodies (B). (C) pRNF114 ubiquitinates NS4B in a dose-dependent manner. HEK293T cells were cotransfected with pMyc-NS4B and increasing concentrations of pFlag-pRNF114 (0, 1, or 2 μg), followed by immunoprecipitation with anti-Myc MAb, and analyzed via immunoblotting with an anti-Ub MAb. (D and E) pRNF114 ubiquitinates NS4B depending on its E3 ubiquitin ligase. Lysates from HEK293T cells which were cotransfected with pMyc-NS4B and pFlag-pRNF114, pRNF114(C64/67A), and pRNF114(69-228), together with pHA-Ub (D) or without pHA-Ub (E), were subjected to immunoblotting followed by co-IP. Ubiquitination of NS4B was detected with anti-HA (D) or anti-Ub (E) MAb.

Protein ubiquitination is a critical step in the proteasome degradation pathway (33). Therefore, we hypothesized that the antiviral potential of pRNF114 could be due to ubiquitination of NS4B. For this purpose, pMyc-NS4B, pHA-Ub, and pFlag-pRNF114 were cotransfected into HEK293T cells. The results showed that NS4B ubiquitination was markedly increased in the presence of pRNF114 (Fig. 8A). We then validated the role of pRNF114 in NS4B ubiquitination in the context of CSFV infection. The results showed that the NS4B ubiquitination was increased in PK-15 cells transfected with pFlag-pRNF114 (Fig. 8B). Next, pMyc-NS4B and increasing doses of pRNF114 (0, 1, or 2 μg) were cotransfected in HEK293T cells, and immunoprecipitation and immunoblotting analyses showed that pRNF114 ubiquitinates NS4B in a dose-dependent manner (Fig. 8C). To determine the role of the E3 ligase activity in the ubiquitination of NS4B, HEK293T cells were cotransfected with pMyc-NS4B and pFlag-pRNF114, pRNF114(C64/67A), or pRNF114(69-228). The ubiquitination analyses revealed that the mutation of the RING domain on pRNF114 reduced NS4B ubiquitination (Fig. 8D). We also detected more endogenous ubiquitin conjugated to NS4B in the presence of pRNF114 but not mutated pRNF114(C64/67A) and pRNF114(69-228) (Fig. 8E). These data suggest that the ubiquitination of NS4B induced by pRNF114 depends on its E3 ligase activity.

### pRNF114 catalyzes the K27-linked polyubiquitination of NS4B.

**FIG 9:**
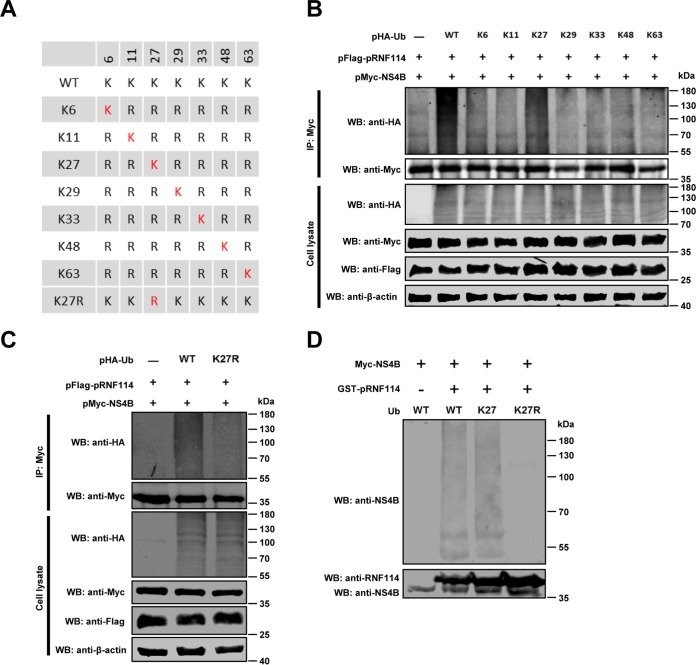
pRNF114 catalyzes K27-linked polyubiquitination of NS4B. (A) Schematic representation of a panel of Ub mutants. (B to D) pRNF114 catalyzes K27-linked NS4B polyubiquitination. HEK293T cells were cotransfected with pMyc-NS4B, pFlag-pRNF114, and pHA-Ub wild types (WT) or mutants (K6, K11, K27, K29, K33, K48, K63, or K27R). Ubiquitination and immunoblotting assays were performed as described above (B and C). An *in vitro* ubiquitination assay was performed according to the instructions of the manufacturer (R&D Systems) (D).

To verify the effects of pRNF114 on the types of polyubiquitination on NS4B, a panel of Ub mutants was included. We first generated an HA-Ub mutant (K0), in which all lysine (K) residues were replaced with arginine (R), and then individual lysine residues (K6, K11, K27, K29, K33, K48, or K63) were reintroduced into the K0 mutant to generate single-lysine mutants (Fig. 9A). pMyc-NS4B and pFlag-pRNF114 together with wild-type HA-Ub or its mutants were transfected into HEK293T cells. The results demonstrated that pRNF114-mediated NS4B polyubiquitination could be detected in the presence of HA-K27-Ub but not of other HA-Ub mutants (Fig. 9B). In contrast, the ubiquitination of NS4B was impaired when using HA-K27R-Ub (HA-Ub with only K27 mutated to R), indicating that pRNF114 mediates the K27-linked polyubiquitination of NS4B (Fig. 9C). Finally, *in vitro* ubiquitination assay further confirmed that pRNF114 mediated K27-linked polyubiquitination of NS4B in the cell-free system (Fig. 9D). These results collectively show that pRNF114 catalyzes K27-linked polyubiquitination of NS4B.

## DISCUSSION

In this study, we clarified the antiviral mechanism of pRNF114, which is involved in interaction with CSFV NS4B and the degradation of NS4B via a proteasome-dependent pathway. We found that pRNF114 efficiently suppressed CSFV growth in PK-15 cells (Fig. 2 and 3). Interestingly, we found that the anti-CSFV effects of pRNF114 depended on its E3 ligase activity (Fig. 4 and 8). Notably, we demonstrated that pRNF114 binds to NS4B and promotes NS4B K27-linked polyubiquitination, leading to the proteasomal degradation of NS4B (Fig. 5 to 8). Taken together, our findings demonstrate that pRNF114 is a novel anti-CSFV protein that acts through targeting NS4B for its degradation.

Our results indicate that pRNF114 inhibits CSFV replication through interacting with NS4B. Since the interaction mediates NS4B degradation, we suppose the disruption of pRNF114 and NS4B would inhibit CSFV replication. It has previously been shown that pRNF114 is involved in T-cell activation (30) and that overexpression of pRNF114 enhances NF-κB and IRF3 activities and increases the expression of type I and III interferons (IFNs). Our findings that pRNF114 directly inhibits CSFV replication have not been previously reported and may contribute to a better understanding of the roles of pRNF114 in antiviral response beyond immunology regulation.

Protein modifications are intimately linked to the protein’s function. It has been demonstrated that ubiquitin-modification regulates the replication of flaviviruses. The ubiquitin ligase CBLL1 and the proteasome-ubiquitin system are required for the cellular internalization of West Nile virus (WNV) and the entry and replication of other flaviviruses (34, 35). The E3 ubiquitin ligase MKRN1 induces degradation of WNV capsid protein in a proteasome-dependent manner (36). It has been reported that the K48-linked polyubiquitination of the DENV NS1 protein inhibits its interaction with the viral partner NS4B, suggesting that NS1 ubiquitination may affect virus replication (37). In addition, it has been shown that the CSFV N^pro^ induces degradation of IRF3 via the proteasome pathway; possibly, N^pro^ recruits E3 ubiquitin ligase complex to degrade IRF3 (38). Since proteasome inhibitor MG132 increases ubiquitination of CSFV NS4B, our study further supports a role for the ubiquitin-proteasome system in flaviviral replication.

The well-understood functions of polyubiquitination are mediated by chains composed of K48 and K63 linkages; however, little is known about the functions of the other noncanonical ubiquitin chains (linked via K6, K11, K27, K29, or K33) (39). Increasing studies suggest that K27-linked polyubiquitination contributes the regulation of proteasomal degradation (40). TRIM40 interacts with RIG-I and MDA5 and promotes their K27/K48-linked polyubiquitination, leading to their proteasomal degradation (41). IpaH9.8, a *Shigella* bacterial ubiquitin E3 ligase, catalyzes K27-linked polyubiquitination of NEMO and undergoes proteasomal degradation (42). Here we demonstrate that the K27-linked polyubiquitination of NS4B is also associated with the ubiquitin-proteasome pathway, providing an additional role for polyubiquitination with K27 linkage. In addition, CSFV NS4B has 23 lysine residues. Six (K48, K63, K178, K206, K258, and K262) lysine residues were predicted to be potential ubiquitination sites by the UbPred program (http://www.ubpred.org/). The specific lysine residues deserve future investigations, which would be extremely valuable in constructing NS4B mutants to support CSFV replication.

Currently, few studies have reported on the interactions between flaviviral NS4B and cellular proteins, possibly due to the lower significance of NS4B in the flavivirus life cycle than of other nonstructural proteins, such as NS3 and NS5. Previous studies have provided clues that the formation of the K27-linked polyubiquitination on target proteins serves as an anchoring platform for its interaction with other functional proteins. Upon microbial DNA challenge, E3 ubiquitin ligase complex INSIG1 and AMFR catalyze the K27-linked polyubiquitination of STING, creating an anchoring platform for recruiting and activating TBK1 (43). TRIM21 positively regulates innate immunity by catalyzing K27-linked polyubiquitination of MAVS and promoting the recruitment of TBK1 to MAVS (44). During DENV replication, the K27-linked polyubiquitination of NS3 is necessary to interact with NS2B to form the NS2B3 protease complex (45). pRNF114 is the first E3 ligase to be identified as being involved in the K27-linked polyubiquitination of the CSFV NS4B, which is a component of the replication complex, together with NS4A, NS3, NS4B, NS5A, and NS5B (7). Therefore, it is possible that this modification will influence the formation of replication complex and, in turn, regulate the replication of CSFV. Our study provides new insights on the role of the host ubiquitin system during CSFV replication. A more comprehensive understanding of this virus-host interplay will be crucial for our understanding of flavivirus pathogenesis and the development of antiviral strategies and treatments.

In summary, here we have reported a novel function of the host E3-ubqiuitin ligase pRNF114 in inhibiting CSFV replication. Because the nonstructural protein NS4B is indispensable for processing the CSFV polyprotein, targeting NS4B could be a crucial route for antiviral development.

## MATERIALS AND METHODS

### Cells and viruses.

HEK293T and PK-15 cells were cultured in Dulbecco’s modified Eagle’s medium supplemented with 10% fetal bovine serum (FBS). PBMCs and PAMs were isolated and maintained in RPMI 1640 medium supplemented with 10% FBS, and all these cells were cultured in a 5% CO_2_ humidified atmosphere. The rCSFV-Fluc strain and the CSFV Shimen strain were propagated in PK-15 cells.

### Construction of plasmids.

The *pRNF114* gene was cloned into the p3×Flag-CMV-10 (Sigma-Aldrich), pFUGW (Addgene), pGEX-6P-1 (GE Healthcare), and pCMV-Myc (Clontech) vectors to generate pFlag-pRNF114, pFUGW-pRNF114, pGST-pRNF114, and pMyc-pRNF114, respectively. To generate site mutants, alanine was substituted for cysteine by PCR. The N- and C-terminal truncation mutants pRNF114(1-68) and pRNF114(69-228) were generated by standard PCR. The *NS4B*, *NS4B(1-179)*, and *NS4B(180-347)* genes were cloned into the pCMV-Myc vector (Clontech) to obtain pMyc-NS4B, pMyc-NS4B(1-179), and pMyc-NS4B(180-347), respectively. The primers for amplification of plasmids are listed in Table 1.

**TABLE 1:** Primers used in this study

Primer	Sequence (5′–3′)	Usage
Flag-pRNF114-F	CCGGAATTCAATGGCGGCCCAACCGCAGGA	Amplification of pRNF114
Flag-pRNF114-R	GGGGTACCTCACTGGTCAAGGAGGGAGC	
Myc-pRNF114-F	CCGGAATTCGGATGGCGGCCCAACCGCAGGA	Amplification of pRNF114
Myc-pRNF114-R	GGGGTACCTCACTGGTCAAGGAGGGAGC	
GST-pRNF114-F	CCGGAATTCATGGCGGCCCAACCGCAG	Amplification of pRNF114
GST-pRNF114-R	GCGTCGACTCACTGGTCAAGGAGGGAG	
Flag-pRNF114(1-68)-F	CGGAATTCAATGGCGGCCCAACCGCAG	Amplification of pRNF114(1-68)
Flag-pRNF114(1-68)-R	CGGGATCCTCAGCGACACACCCCACA	
Flag-pRNF114(69-228)-F	CGGAATTCAATGAGCACTCTGGCCCCTG	Amplification of pRNF114(69-228)
Flag-pRNF114(69-228)-R	CGGGATCCTCACTGGTCAAGGAGGGAG	
Flag-pRNF114(C64/67A)-F	CCGAAGAAGCCTGTCGCTGGGGTGGCTCGCAGCACTCTGGCC	Amplification of pRNF114(C64/67A)
Flag-pRNF114(C64/67A)-R	GGCCAGAGTGCTGCGAGCCACCCCAGCGACAGGCTTCTTCGG	
pRNF114(NGG mut)-F	ACAGGCCATTACGGCCATGGCGGCCCAACCGCAGGATCGAGAAGG	Amplification of pRNF114(NGG mut)
pRNF114(NGG mut)-F	TACGGCCGAGGCGGCCTTATCACTGGTCAAGGAGGGAGCGTTG	
Myc-NS4B-F	CGGAATTCATGCTCAGGGGGATGTGCA	Amplification of NS4B
Myc-NS4B-R	CCGCTCGAGTTATAGCTGGCGGATCTTTCC	
Myc-NS4B(1-179)-F	CGGAATTCATGCTCAGGGGGATGTGCA	Amplification of NS4B(1-179)
Myc-NS4B(1-179)-R	GGGGTACCTTATAGTTTGAGAGCTGTGGCGGCA	
Myc-NS4B(180-347)-F	CCGGAATTCGGTTCGCCCCCACACGATTGGAGAG	Amplification of NS4B(180-347)
Myc-NS4B(180-347)-R	GGGGTACCTTATAGCTGGCGGATCTTTC	
Flag-NS4B-F	CCGGAATTCAATGGCTCAGGGGGATGTGCAGA	Amplification of NS4B
Flag-NS4B-R	GGGGTACCTTATAGCTGGCGGATCTTTC	
Q-NS4B-F	AGACAGTCGGCAACCCT	RT-qPCR for detection of NS4B
Q-NS4B-R	AGCCTCGAACATTATCAAA	
Q-pRNF114-F	GACACGTCTTTTGCTCTGCATG	RT-qPCR for detection of pRNF114
Q-pRNF114-R	TGGCAAGAAGTCTCTGTGCTCTC	
Q-GAPDH-F	GAAGGTCGGAGTGAACGGATTT	RT-qPCR for detection of GAPDH
Q-GAPDH-R	TGGGTGGAATCATACTGGAACA	

### Generation of PK-pRNF114 and PK-pRNF114(C64/67A) cells.

The recombinant plasmids pFUGW-pRNF114 and pRNF114(C64/67A) were constructed, and the recombinant lentiviruses (MOI = 1), including Lenti-pRNF114, Lenti-pRNF114(C64/67A), and Lenti-EGFP (control), were transduced into PK-15 cells; these cells were separated by flow cytometry twice.

### Generation of PK-RNF114-KO cells.

To generate PK-RNF114-KO cells, the single guide RNA (sgRNA) was designed using an online CRISPR design tool (http://crispr.mit.edu/) to target exons 1 and 2 of pRNF114 (Table 2). The lentiCRISPRv2 plasmid was digested using BsmBI (New England BioLabs [NEB]), and pCRISPRv2-sgRNA-RNF114-1 and pCRISPRv2-sgRNA-RNF114-2 were constructed. All of the constructs in this study were verified by sequencing. PK-15 cells were transduced and selected in the presence of 2.5 μg/ml of puromycin (Solarbio) and seeded on 96-well plates with serial dilutions to obtain a single-cell-derived colony. Two weeks later, the cells were collected and genomic DNA was extracted; the *pRNF114* gene sequence was detected using PCR and sequencing analysis.

**TABLE 2:** sgRNAs used in this study

sgRNA	Sequence	
	Sense (5′–3′)	Antisense (5′–3′)
sgRNA-RNF114-1	CACCGGGCCCAACCGCAGGATCGAG	AAACCTCGATCCTGCGGTTGGGCCC
sgRNA-RNF114-2	CACCGAATCTGCCGCTCGAGCTCCA	AAACTGGAGCTCGAGCGGCAGATTC

### Cell viability assay.

Cell viability was determined by using Cell Counting Kit-8 (CCK-8; Dojindo) according to the manufacturer’s instructions.

### IFA.

Culture medium was collected and measured for titers of CSFV by indirect immunofluorescence assay (IFA) as reported previously (46).

### Real-time RT-qPCR.

Total RNA extracted from CSFV-infected PK-15 cells or other cells was treated with TRIzol reagent (Invitrogen) and was reverse transcribed into the cDNA using avian myeloblastosis virus (AMV) reverse transcriptase XL (TaKaRa). Viral RNA copies were quantified using a previously described RT-qPCR assay (47).

The transcription levels of RNF114 and NS4B in PK-15 cells with or without Shimen infection were quantified by the threshold cycle (2^−ΔΔ^*^CT^*) method (48). The mRNA level of glyceraldehyde-3-phosphate dehydrogenase (GAPDH) was set as an internal loading control. The primers used for real-time RT-PCR are listed in Table 1.

### Western blotting and co-IP assay.

Samples were lysed with NP-40 lysis buffer containing a protease inhibitor (1 mM phenylmethylsulfonyl fluoride [PMSF]; Beyotime) for 30 min and then centrifuged for 20 min at 4°C; the supernatants were denatured in 5× sample loading buffer for 7 min at 100°C. For immunoprecipitation assays, the supernatants were mixed with the respective primary antibodies in the presence of protein G-agarose (Roche) at 4°C overnight, followed by Western blotting.

### GST pulldown assay.

GST pulldown was performed as described previously (46). Briefly, purified GST or GST-pRNF114 fusion protein was mixed with lysates from cells transfected with pMyc-NS4B at 4°C overnight. The proteins pulled down by GST-agarose were detected by Western blotting using a rabbit anti-Myc monoclonal antibody (MAb) and a mouse anti-GST polyclonal antibody (PAb) (Tiangen).

### Confocal imaging.

The cells were seeded in glass-bottom dishes. HEK293T cells were cotransfected with the pFlag-pRNF114 and pMyc-NS4B (1 μg each) for 36 h. PK-pRNF114 cells were infected with Shimen at an MOI of 1 for 48 h. Then the cells were stained with the relevant antibodies and images were acquired by using a Leica SP2 confocal system (Leica Microsystems) (31).

### Ubiquitination assay.

pMyc-NS4B and pHA-Ub were cotransfected into HEK293T cells with or without pFlag-pRNF114. The cells were then treated with 10 μM MG132 (Sigma-Aldrich) for 24 h and were lysed by NP-40 lysis buffer containing a deubiquitinase inhibitor (10 μM *N*-ethylmaleimide [NEM]; Sigma-Aldrich). Then the anti-Myc MAb-conjugated agarose beads were added into the samples separately. Following incubation overnight at 4°C, the samples were examined by Western blotting.

For the *in vitro* ubiquitination assay, bacterially expressed GST fusion proteins of pRNF114 and pRNF114(C64/67A) were captured on glutathione-Sepharose beads and incubated for 90 min at 30°C with purified Ub-activating enzyme UBE1, His-tagged Ub-conjugating enzyme UbcH5A, Flag-Ub, and Ub conjugation reaction buffer (R&D Systems). The ubiquitin ligase activity of pRNF114 was analyzed by Western blotting with an anti-Flag MAb. For *in vitro* ubiquitination of NS4B by pRNF114, purified NS4B and pRNF114 were incubated with UBE1, UbcH5A, His-Ub, His-K27, or His-K27R in the presence of ATP. The *in vitro* ubiquitination of NS4B was analyzed by Western blotting.

### Statistical analysis.

All experiments were performed at least three times. Data were statistically analyzed by Student’s *t* test using SPSS 22.0 software. Differences were considered significant if the unadjusted *P* value was less than 0.05.
